# Quality of Life Among Family Caregivers of Individuals With Rare Diseases: Web-Based Population Study on the Validity and Reliability of the Polish World Health Organization Quality of Life-BREF Questionnaire

**DOI:** 10.2196/72590

**Published:** 2025-07-18

**Authors:** Piotr Jabkowski, Jan Domaradzki, Dariusz Walkowiak

**Affiliations:** 1 Faculty of Sociology, Adam Mickiewicz University, Poznan, Poland; 2 Department of Social Sciences and Humanities, Poznan University of Medical Sciences, Rokietnicka 7, Poznań, 60-806, Poland, 48 695324630; 3 Department of Organization and Management in Health Care, Poznan University of Medical Sciences, Poznan, Poland

**Keywords:** quality of life, rare diseases, caregivers, WHOQOL-BREF, World Health Organization Quality of Life-BREF, validation

## Abstract

**Background:**

Caring for individuals with rare diseases (RDs) presents unique challenges that can significantly impact caregivers’ quality of life (QoL). The World Health Organization Quality of Life-BREF (WHOQOL-BREF) is a widely used tool for assessing QoL across different populations.

**Objective:**

This study examines the QoL of caregivers of individuals with RDs and evaluates the psychometric properties of the WHOQOL-BREF in this population.

**Methods:**

A self-administered, anonymous, computer-assisted web-based survey was conducted among family caregivers of individuals with RDs in Poland between March and August 2024. Due to the lack of a national registry of patients with RDs, participants were recruited through convenience sampling via associations, foundations, and organizations of patients with RDs. Eligibility criteria included being 18 years and older of age, speaking Polish, being a caregiver of a person with a confirmed RD diagnosis, and providing informed consent. The survey included sociodemographic questions and the Polish version of the WHOQOL-BREF, which assesses QoL across 4 domains: physical health, psychological health, social relationships, and environment. Internal consistency was assessed using Cronbach α, and confirmatory factor analysis was conducted to examine the instrument’s structural validity.

**Results:**

A total of 942 caregivers of individuals with various RDs participated in the study. Confirmatory factor analysis supported the 4-domain structure, with further improvement in a finally modified WHOQOL-BREF model (*χ*^2^_243_=1043.0; *P*<.001; Comparative Fit Index=0.919; Tucker-Lewis Index=0.907; root-mean-square error of approximation=0.059). Internal consistency was satisfactory, with Cronbach α values ranging from 0.70 (social relationships) to 0.84 (psychological health). Mean domain scores on a scale of 0‐100 were 50.2 (SE 0.59; physical health), 54.9 (SE 0.59; psychological health), 51.3 (SE 0.67; social relationships), and 52.1 (SE 0.56; environment), with minimal floor and ceiling effects (≤1.4%) across domains. Younger female caregivers reported significantly lower psychological health (eg, mean 43.6, SE 0.59 vs mean 59.9, SE 10.0 for younger male caregivers) and social relationships (mean 39.3, SE 3.34 vs mean 55.0, SE 4.75) well-being compared to other groups. Exactly 151 (16%) of caregivers rated their overall QoL as poor or very poor, and 289 (30.7%) were dissatisfied or very dissatisfied with their health, with female caregivers reporting more dissatisfaction (n=263, 32.4%) than male caregivers (n=26, 20%). Overall, our findings demonstrate the robust psychometric properties of the WHOQOL-BREF among caregivers of people with RDs and provide domain-specific normative data to guide future research and interventions.

**Conclusions:**

The WHOQOL-BREF is a reliable and valid instrument for assessing QoL among caregivers of individuals with RDs, though the social relationship domain may require further refinement. Caregivers experience varying QoL outcomes depending on demographic factors, highlighting the need for targeted support interventions. Future research should explore cultural adaptations and potential supplementary modules to enhance the instrument’s applicability in caregiver populations.

## Introduction

Quality of life (QoL) is a multidimensional construct that reflects an individual’s overall well-being, encompassing physical health, psychological health, social relationships, and environmental factors. In 1991, the World Health Organization (WHO) initiated a project to develop an internationally applicable measure of QoL, leading to the creation of the World Health Organization Quality of Life (WHOQOL) instrument. The WHO defined QoL as an individual’s perception of their position in life within the context of their cultural and value systems as well as their personal goals, expectations, standards, and concerns. This definition highlights the subjective nature of QoL, emphasizing that it is shaped by personal experiences and influenced by broader social and environmental contexts. Importantly, QoL assessment provides valuable insights into health outcomes, treatment effectiveness, and overall well-being across diverse populations [1-3].

A subset of QoL research, health-related quality of life (HRQoL), has gained increasing attention in clinical and public health research. HRQoL focuses specifically on the impact of health conditions on an individual’s well-being, playing a critical role in health care decision-making, policy development, and patient-centered care [4-6]. Over the past 3 decades, numerous HRQoL assessment tools have been developed, including both generic instruments applicable across populations and disease-specific measures tailored to particular conditions. Despite the proliferation of these tools, a significant limitation remains—the lack of internationally validated instruments capable of capturing HRQoL across diverse cultural and socioeconomic settings [7,8].

To address this challenge, the WHO developed the WHOQOL-100 consisting of 100 questions, an instrument designed to assess subjective well-being while ensuring cross-cultural validity. This instrument was developed through extensive research conducted across 15 field centers worldwide, incorporating perspectives from patients, caregivers, health care professionals, and the general population. The psychometric properties of the WHOQOL-100, including its validity and reliability, were rigorously tested in multiple cultural and linguistic contexts [1]. Recognizing the need for a shorter, more practical instrument, the WHO subsequently introduced the World Health Organization Quality of Life-BREF (WHOQOL-BREF) in 1998, a 26-item version that assesses 4 key domains of HRQoL: physical health, psychological health, social relationships, and environment. The WHOQOL-BREF has since been widely adopted in epidemiological studies, clinical trials, and public health research, demonstrating strong psychometric properties and cross-cultural applicability [2,4,7,9].

Although the WHOQOL-BREF has been validated in numerous populations, its application in certain groups remains underexplored. In Poland, the WHOQOL-BREF was validated in 2006 using a sample of healthy individuals and patients with various diseases and health conditions [6]. However, this validation process overlooked caregivers, an essential yet often neglected population in HRQoL research. Caregivers play a vital role in the health care system, providing continuous support to individuals with chronic illnesses and disabilities. Despite their crucial contributions, caregivers often experience significant physical, emotional, and financial burdens, which in turn affect their own QoL [10-13].

Caregivers of persons with RDs face particularly severe challenges. RDs are often characterized by delayed diagnoses, limited treatment options, and a lack of specialized health care resources, placing an immense strain on families [10,13-17]. Studies suggest that caregivers of persons with RDs report lower QoL compared to those caring for individuals with more common chronic conditions, mainly due to increased caregiving demands, emotional distress, social isolation, and financial hardship. The unpredictable nature of many RDs, coupled with the complexity of care required, exacerbates stress and negatively affects the physical and mental health of caregivers [5,13,18].

The burden of caregiving is multifaceted, affecting various domains of QoL. Physically, caregivers frequently experience chronic fatigue, sleep disturbances, headaches, and weakened immune function due to the demands of continuous care. Psychologically, they are at increased risk for anxiety, depression, burnout, and emotional exhaustion, with some studies indicating higher rates of suicidal ideation among caregivers [17-20]. Socially, caregivers often face reduced participation in social activities, strained relationships, and isolation, as caregiving responsibilities limit their ability to engage with friends and family. Financially, many caregivers are forced to reduce work hours or leave employment altogether, exacerbating economic difficulties and increasing reliance on social support systems. Given these profound challenges, assessing QoL in caregivers of persons with RDs is essential for understanding their specific needs and designing effective interventions to support them [14,16,19,21-23].

Despite the growing recognition of caregiver burden as a public health concern, research validating HRQoL assessment tools specifically for this population remains limited. The WHOQOL-BREF, although widely used across various patient populations, has not been systematically evaluated for its appropriateness in assessing the QoL of caregivers, particularly in the context of rare diseases (RDs). While WHOQOL-BREF is often used in studies on caregivers of individuals with RDs, the resulting QoL domain scores are typically interpreted abstractly—detached from the scale itself—or, less commonly, compared against national normative data [17,24,25]. A validated, standardized tool tailored to caregivers would offer valuable insights for clinical practice, inform health policy, and support the development of targeted interventions, ensuring that the specific needs of caregivers are effectively addressed [2,13,25,26].

To bridge this research gap, this study aims to validate the WHOQOL-BREF for caregivers of persons with RDs and establish population norms specific to this group. By generating normative data, this study seeks to enhance the clinical utility of the WHOQOL-BREF in caregiver research and contribute to the development of targeted support programs and health care policies. A comprehensive understanding of caregiver QoL is crucial for improving the well-being of both caregivers and the care recipients they support, ultimately leading to better health outcomes for families affected by RDs.

## Methods

### Study Design

A cross-sectional study was conducted in 2024 in accordance with the STROBE (Strengthening the Reporting of Observational Studies in Epidemiology) guidelines (Checklist 1) [27].

This study used data from a self-administered, anonymous, computer-assisted web-based survey examining the association between caregiving for a person with an RD and caregivers’ perceived QoL. The survey targeted family caregivers of individuals with RDs. Given the absence of a Polish registry of patients with RD and the unknown exact number of affected individuals, a convenience sampling approach was used. Several associations, foundations, and organizations of patients with RD assisted in participant recruitment by sharing the survey link on their websites and Facebook pages.

At the same time, it should be stressed that convenience sampling used in this study introduces selection bias, as participants may not represent the broader population, limiting the generalizability of findings. However, given the specific nature of the study population—namely, the small size of the groups of particular RDs, the absence of a national registry of patients with RD in Poland, and the wide geographic dispersion of caregivers—convenience sampling appeared to be the most practical and, indeed, the only feasible method. However, it is important to acknowledge several limitations associated with this approach. Most importantly, participants recruited via patient organizations may not reflect the full diversity of the broader population. This type of sampling could affect the study outcomes, potentially skewing results based on the demographics of the sample, such as age, sex, socioeconomic status, or disease severity. Consequently, this method introduces selection bias and limits the reliability and generalizability of the findings. Thus, although this strategy was appropriate under the circumstances, future research should aim to use more representative sampling techniques—such as random or stratified sampling—that may better capture the heterogeneity of caregivers of individuals with RDs and allow for a more accurate reflection of the realities of the population as a whole. Nonetheless, again, it should be stressed that due to the unique challenges of studying this population of caregivers of individuals with RDs, the use of other sampling methods is very difficult, if at all feasible.

### Setting and Recruitment

The survey targeted family caregivers of individuals diagnosed with RDs. Due to the absence of a national registry of patients with RD in Poland and the unknown exact number of patients with RD, the study used convenience sampling. Recruitment was supported by various patient organizations, foundations, and advocacy groups via their websites and Facebook profiles.

At the first stage of recruitment, the study coordinator contacted several associations, foundations, and organizations of patients with RD to determine whether they were interested in participating in the study. After receiving their consent, a letter with detailed information about the purpose and method of the study, together with an invitation, was published on the organization’s Facebook pages and sent out via an internal mailing to members, by which all affiliated caregivers who met the inclusion criteria were invited to participate in the study.

Eligible participants were required to be at least 18 years of age, fluent in Polish, and a parent or family caregiver of a person with a confirmed RD diagnosis. Additionally, participants needed access to the internet, the ability to use electronic devices to complete the survey, and had to provide written informed consent before participation.

The questionnaire consisted of sociodemographic questions concerning both caregivers and persons with RD along with the abbreviated Polish version of the WHOQOL-BREF [6]. This 24-item instrument assesses QoL across 4 domains: physical health, psychological health, social relationships, and environment. Two additional items do not constitute any of the 4 dimensions but are set up to measure the overall QoL and satisfaction with health.

### Data Collection Procedure

The survey was conducted between March and August 2024. Initially, the research coordinator contacted multiple associations, foundations, and organizations of patients with RDs to obtain permission for distribution. Upon approval, an invitation letter containing a link to the web-based questionnaire was shared via these platforms. Participants who provided written informed consent completed the survey electronically. To enhance response rates, 3 follow-up reminders were sent in January, March, and June. The survey required approximately 20‐25 minutes to complete.

### Data Analysis

All statistical analyses were conducted using R Statistical Software (version 4.3.1; R Foundation for Statistical Computing). The study used a quantitative research design to analyze the psychometric properties of the WHOQOL-BREF questionnaire for the Polish population of caregivers of people with RDs. Descriptive statistics were calculated to summarize the demographic characteristics of the sample and the overall QoL scores in 4 domains: physical health, psychological health, social relationships, and environment. Reliability analysis was performed using Cronbach α to assess the internal consistency of the questionnaire. Confirmatory factor analysis (CFA) was used to test the hypothesized factor structure of the WHOQOL-BREF by examining the fit of the data to the model. Goodness-of-fit indices, including the Comparative Fit Index (CFI) and root mean-square-error of approximation (RMSEA), were reported to assess model adequacy.

### Ethical Considerations

This study was conducted according to the Declaration of Helsinki and was approved by the Poznan University of Medical Sciences Bioethics Committee (KB 228/24, March 13, 2024). All participants were informed about the objectives and methodology of the study in an invitation letter. Informed consent was obtained electronically through the web-based questionnaire by selecting the “I agree” option. Participants were assured that their participation was voluntary, anonymous, and confidential and that they had the right to withdraw at any time or omit any question without consequence. To ensure privacy, no personal identifying information or images were collected. Participants were also informed that, although some questions may be emotionally sensitive, they could pause or discontinue the survey at any time. Since participation did not involve any direct intervention or additional burden, no financial compensation was offered.

## Results

### Overview

In this section, we present a validation of the WHOQOL-BREF scale and provide insights into its empirical structure, psychometric properties, and latent score characteristics. We have implemented CFA by comparing a unidimensional baseline model with the established 4D model of the WHOQOL-BREF. Statistical fit indices such as CFI and RMSEA are provided to assess model adequacy, along with modifications to improve dimensional alignment. This section then highlights the robustness of the 4D structure, and the Structural Validity section focuses on the reliability of the WHOQOL-BREF scale, which is examined through the lens of internal consistency across the 4 domains. A descriptive analysis of the scores derived from the 4 dimensions of the WHOQOL-BREF is also presented. Finally, norms for the QoL domains in a Polish population of caregivers of persons with RDs are provided. The norms for the WHOQOL-BREF are presented as mean values, SE of the mean, 25th percentile, median, 75th percentile, minimum, maximum, and percentage of floor and ceiling effect. These norms were estimated by sex and age group.

### Study Participants

Table 1 presents characteristics of the sample by sex and age category of caregivers.

Table 2 outlines the demographic and diagnostic characteristics of 942 patients with RDs. Most patients were male (n=586, 62.2%), with a wide age range (0.2‐78 years), a mean age of 12 (SE 0.32) years, and a median of 10 (IQR 6.0-15.0) years. The most frequently reported conditions included Duchenne muscular dystrophy or Becker muscular dystrophy (n=211, 22.4%), 22q11.2 deletion syndrome (n=131, 13.9%), and Angelman syndrome (n=120, 12.7%). A total of 191 (20.3%) patients were diagnosed with one of 144 other rare or ultrarare conditions. Time to diagnosis varied considerably: while 165 (17.5%) were diagnosed within 3 months, 226 (24%) received a diagnosis within 1‐2 years, and 130 (13.9%) waited more than 5 years. A small group (n=18, 1.9%) was still in the asymptomatic phase of the disease. These data highlight the diagnostic challenges and heterogeneity of RD populations.

**Table 1.** General characteristics of family caregivers of individuals with rare disease.

Age (years)	Male, n (%)	Female, n (%)	Total, n (%)
16‐29	2 (1.5)	32 (3.9)	34 (3.6)
30‐39	44 (33.8)	337 (41.5)	381 (40.4)
40‐49	64 (49.2)	350 (43.1)	414 (43.9)
50 and more	20 (15.4)	93 (11.5)	113 (12.0)
Total	130 (13.8)	812 (86.2)	942 (100)

**Table 2.** Characteristics of individuals with rare disease.

Characteristics	Total (N=942), n (%)
Sex	
Female	356 (37.8)
Male	586 (62.2)
Age (years)	
Mean (SE)	12 (0.32)
Range	0.2‐78
Median (IQR)	10 (6.0-15.0)
Disease	
22q11.2 deletion syndrome	131 (13.9)
Angelman syndrome	120 (12.7)
DMD^a^ or BMD^b^	211 (22.4)
Dravet syndrome	106 (11.3)
Ehlers-Danlos syndrome	19 (2)
Glycogen storage disease	10 (1.1)
Kabuki syndrome	11 (1.2)
Neurofibromatosis	33 (3.5)
Niemann-Pick disease	10 (1.1)
PANS^c^ or PANDAS^d^	14 (1.5)
Prader-Willi syndrome	10 (1.1)
Tuberous sclerosis complex	14 (1.5)
Turner syndrome	14 (1.5)
Williams syndrome	48 (5.1)
144 other diseases	191 (20.3)
Time spent before the correct diagnosis was made	
0‐3 months	165 (17.5)
4‐6 months	106 (11.3)
7‐12 months	140 (14.9)
1‐2 years	226 (24.0)
3‐5 years	157 (16.7)
6‐10 years	82 (8.7)
11‐15 years	29 (3.1)
16‐20 years	10 (1.1)
20+ years	9 (1.0)
Asymptomatic phase of the disease	18 (1.9)

a DMD: Duchenne muscular dystrophy.

b BMD: Becker muscular dystrophy.

c PANS: pediatric acute-onset neuropsychiatric syndrome.

d PANDAS: pediatric autoimmune neuropsychiatric disorders associated with streptococcal infection.

### Confirmatory Factor Analysis

The CFA results illustrate a comparative assessment of 3 models for the WHOQOL-BREF: the baseline unidimensional model, the original 4D WHO model, and a modified 4D model. The fit indices provide a comprehensive evaluation of the models’ performance and structural validity of the WHOQOL-BREF 4D scale (Table 3).

The baseline unidimensional model yielded unsatisfactory fit indices, suggesting a poor representation of the scale’s underlying structure. The chi-square statistic (*χ*^2^_252_=1602.8; *P*<.001) was significant, and the *χ*^2^/*df* ratio (6.360) exceeded the acceptable threshold of 5. The CFI (0.862) and Tucker-Lewis Index (TLI=0.849) indicated a suboptimal model fit, while the RMSEA (0.075; 90% CI 0.072‐0.079) was marginally above the acceptable upper limit. Conversely, the standardized root-mean-square residual (SRMR=0.050) remained within acceptable limits, indicating an adequate residual-based fit. In practical terms, these results suggest that treating the WHOQOL-BREF as a single-factor measure does not fully capture the complexities of caregivers’ QoL, as evidenced by the modest CFI or TLI and moderately elevated RMSEA.

**Table 3.** Comparison of fit indices for different WHOQOL-BREF^a^ latent models for family caregivers of individuals with rare disease.

Model	Chi-square (*df*)	*χ*^2^/*df*	CFI^b^	TLI^c^	SRMR^d^	RMSEA^e^	RMSEA−95% CI	RMSEA+95% CI
Baseline model	1602.8 (252)	6.4	0.862	0.849	0.050	0.075	0.072	0.079
Original WHOQOL-BREF	1343.9 (246)	5.5	0.888	0.875	0.047	0.069	0.065	0.072
Modified WHOQOL-BREF	1043.0 (243)	4.3	0.919	0.907	0.041	0.059	0.055	0.063

a WHOQOL-BREF: World Health Organization Quality of Life-BREF.

b CFI: Comparative Fit Index.

c TLI: Tucker-Lewis Index.

d SRMR: standardized root-mean-square residual.

e RMSEA: root-mean-square error of approximation.

In contrast, the initial 4D model demonstrated substantial improvements across all fit indices (*χ*^2^_246_=1343.9; *P*<.001). The *χ*^2^/*df* ratio decreased to 5.463, and the CFI (0.888) and TLI (0.875) approached recommended levels. The RMSEA (0.069; 90% CI 0.065‐0.072) and SRMR (0.047) further suggested enhanced fit. Adjustments were made based on modification indices to optimize model fit; that is, correlated error terms were introduced between questions 3 and 4 (modification index equals 164.9), questions 12 and 23 (modification index equals 64.8), and questions 19 and 20 (modification index equals 56.4). While these modifications enhanced statistical compatibility, they also entailed conceptual implications. Specifically, it can be posited that each of these item pairs is likely to tap into closely related dimensions of caregiver well-being (eg, physical or psychological strain or environmental or social considerations). Consequently, the modification indices suggest that some questions, though intended to measure slightly different constructs, may elicit overlapping responses from caregivers who face similar daily challenges across multiple domains of life. Rather than perceiving these errors as mere statistical artifacts, it is more fruitful to interpret them as potential indicators of content overlap or a shared contextual factor (eg, stress or time pressure) that influences the way participants interpret these items. For researchers using the WHOQOL-BREF in caregiver populations, acknowledging and examining these correlated errors can foster a more nuanced understanding of how caregiving experiences merge otherwise distinct QoL facets. Future studies could investigate whether such overlaps are unique to certain subgroups or care contexts or if item wording could be refined to differentiate better between seemingly related aspects of QoL.

The modified 4D model demonstrated a significant enhancement (*χ*^2^_243_=1043.0; *P*<.001), exhibiting a reduced *χ*^2^/*df* ratio of 4.292. The CFI (0.919) and TLI (0.907) exceeded the conventional threshold for adequate fit, while the RMSEA (0.059; 90% CI 0.055‐0.063) and SRMR (0.041) substantiated the model’s robustness. The findings suggest that the targeted modifications serve to enhance the correlation between items within their respective dimensions, thereby producing a more robust and precise measurement structure with fit indices that approach commonly accepted benchmarks.

### Structural Validity

This section presents a reliability analysis of the items within the WHOQOL-BREF across its 4 dimensions. The analysis uses Cronbach α to evaluate internal consistency, with findings indicating satisfactory to high reliability across domains. Specifically, α values ranged from 0.69 for the social relationships domain to 0.84 for the psychological health domain, demonstrating varying but overall strong consistency (Table 4).

Item-level analyses provide further insight into the impact of individual items on the overall reliability of each domain. Most items contribute positively to reliability, with suggestions for potential refinement in some areas.

**Table 4.** Internal consistency scores of the 4 WHOQOL-BREF domains obtained for Polish family caregivers of individuals with rare diseases.

Domain	Items, n	Cronbach α
Physical health	7	0.818
Psychological health	6	0.843
Social relationships	3	0.700
Environment	8	0.807

a WHOQOL-BREF: World Health Organization Quality of Life-BREF.

### Normalized Scores for Domains of WHOQOL-BREF

This section presents a descriptive analysis of the normalized scores derived from the 4D-modified WHOQOL-BREF scale. Following the recommendations of the WHO, responses to each item were coded from 1 to 5 and transformed to a scale from 0 (lowest QoL) to 100 (highest QoL). Each item was weighted by standardized factor loadings derived from a CFA model before calculating the overall domain score (Table 5).

The factor loadings range from 0.363 to 0.801, indicating varying degrees of item contribution to their respective latent constructs. The physical health domain exhibits robust item representation, with loadings such as question 18 (0.801) and question 16 (0.794) suggesting substantial explanatory power for the physical health construct. The psychological health domain also exhibits high loadings, particularly for question 26 (0.747) and question 19 (0.688), suggesting that these items are key indicators of psychological health. Items in the environmental domain display more variability, with question 12 having the lowest loading (0.456) and question 8 the highest (0.763). The social relationships domain demonstrates moderate item loadings, with question 22 (0.619) being the most influential item and question 20 (0.477) contributing the least.

**Table 5.** Standardized factor loadings for the items comprising the 4 WHOQOL-BREF domains for Polish family caregivers of individuals with rare diseases.

WHOQOL-BREF domains and items		Standardized factor loadings
Physical health		
	Question 3	0.456
	Question 4	0.363
	Question 10	0.794
	Question 15	0.673
	Question 16	0.562
	Question 17	0.801
	Question 18	0.658
Psychological health		
	Question 5	0.747
	Question 6	0.689
	Question 7	0.600
	Question 11	0.631
	Question 19	0.795
	Question 26	0.688
Social relationships		
	Question 20	0.776
	Question 21	0.603
	Question 22	0.619
Environment		
	Question 8	0.763
	Question 9	0.677
	Question 12	0.622
	Question 13	0.564
	Question 14	0.675
	Question 23	0.477
	Question 24	0.395
	Question 25	0.380

a WHOQOL-BREF: World Health Organization Quality of Life-BREF.

**Figure 1.**
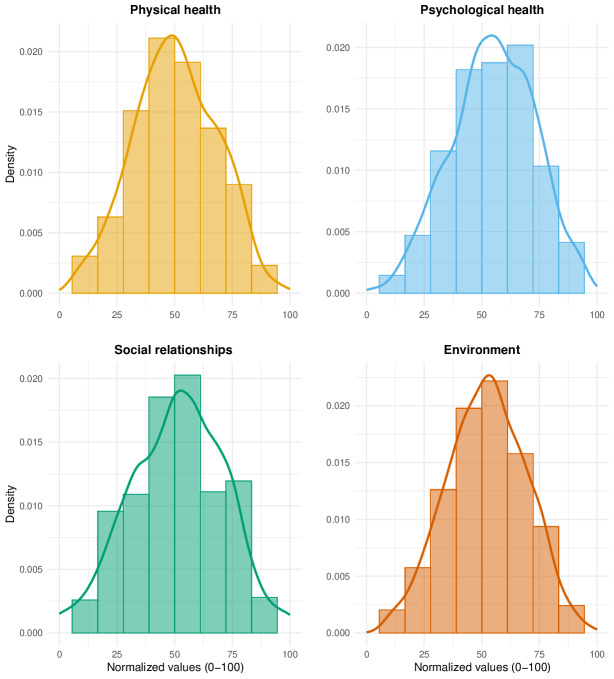
Histogram and density plots for domains of the WHOQOL-BREF (World Health Organization Quality of Life-BREF) for Polish family caregivers of individuals with rare disease.

Figure 1 presents histograms and density plots for the 4 dimensions of the WHOQOL-BREF scale: physical health, psychological health, social relationships, and environment domains. Each plot illustrates the distribution of normalized scores (ranging from 0 to 100) for the respective dimension. The overlaid density curves provide a smoothed visualization of the score distribution, highlighting its approximate normality across all domains, with slight deviations observed, particularly in the psychological health and social relationships domains. The detailed descriptive statistics for the distribution of scores within each domain of the WHOQOL-BREF are delineated in Table 6.

The psychological health domain demonstrated the highest mean score (mean 54.9, SE 0.59), indicating relatively better perceived psychological well-being among respondents. Conversely, the physical health domain exhibited the lowest mean score (mean 50.2, SE 0.59). The environment and social relationships domains showed mean scores of 52.1 (SE 0.56) and 51.3 (SE 0.67), respectively. The SEs of the mean were relatively consistent, ranging from 0.56 to 0.67, indicating precision in the central tendency estimates.

Concerning the normality of the data, the skewness and kurtosis values are within acceptable ranges, thereby supporting the visual interpretation from the density plots. However, the Shapiro-Wilk test reveals statistically significant deviations from normality for the physical health, psychological health, and social relationships dimensions (*P*<.01), while the environment domain demonstrates borderline nonsignificance (*P*=.05), suggesting a near-normal distribution.

**Table 6.** Descriptive characteristics of the latent scores for WHOQOL-BREF domains for Polish family caregivers of individuals with rare disease.

Statistic	Environment	Physical health	Psychological health	Social relationships
Mean (SE)	52.1 (0.56)	50.2 (0.59)	54.9 (0.59)	51.3 (0.67)
Range	6.8-97.8	4.8-100.0	0.0-100.0	0.0-100.0
Skewness	−0.060	−0.023	−0.155	−0.137
Kurtosis	2.624	2.551	2.629	2.681
Shapiro-Wilk statistic	0.997	0.995	0.994	0.989
Shapiro-Wilk *P* value	.05	.006	<.001	<.001
Median (IQR)	52.2 (40.4-64.5)	50.0 (37.8-63.9)	54.6 (42.6-67.2)	51.9 (34.9-67.3)
Floor effect (%)	0	0	0.1	1.4
Ceiling effect (%)	0	0.1	0	1.3

a WHOQOL-BREF: World Health Organization Quality of Life-BREF.

The analysis of floor and ceiling effects reveals minimal clustering at the extremes, with the social relationships domain exhibiting a 1.4% floor effect and a 1.3% ceiling effect, the highest among all domains. These negligible effects ensure the scale’s capacity to capture a wide range of responses without response saturation.

### Normative Data for Domains of WHOQOL-BREF

Prior to the presentation of normative data for the domains of the WHOQOL-BREF, an examination of the overall assessment of the QoL and satisfaction with health among Polish caregivers of individuals with RDs is warranted. The data presented in Table 7 reveal notable differences by sex.

Concerning the overall evaluation of QoL, the predominant response among male and female caregivers was “good,” with 42.3% (n=55) of male and 39.5% (n=321) of female caregivers indicating this as their response. The second most prevalent response was “neither poor nor good,” with 31.5% (n=41) of male and 39.5% (n=321) of female caregivers choosing this option. However, a higher proportion of female caregivers reported a “poor” QoL (n=108, 13.3%) compared to male caregivers (n=19, 14.6%). The percentage of caregivers who rated their QoL as “very poor” was relatively low overall, at 2.5% (n=24), with male caregivers (n=7, 5.4%) more likely to report this than female caregivers (n=17, 2.1%). Conversely, only a small percentage of caregivers reported a “very good” QoL (male: n=8, 6.2% and female: n=45, 5.5%). These findings suggest that perception of the overall QoL is more likely to be situated toward the middle or lower end of the scale for both sexes. Satisfaction with health, however, revealed more pronounced differences between male and female caregivers. While 45.4% (n=59) of male caregivers reported being “satisfied” with their health, this proportion was significantly lower for female caregivers, at 29.2% (n=237). Instead, more female caregivers expressed dissatisfaction (n=237, 29.2% compared to n=22, 16.9% of male caregivers) or were “neither satisfied nor dissatisfied” (female: n=293, 36.1% vs male: n=41, 31.5%). A small proportion of caregivers described themselves as “very satisfied” with their health (male: n=4, 3.1% and female: n=19, 2.3%), while 3.2% (n=30) of caregivers overall reported being “very dissatisfied.” These results suggest that male caregivers generally report a higher QoL and greater health satisfaction than female caregivers. The elevated incidence of dissatisfaction and neutral responses among female caregivers may indicate the supplementary burdens they encounter in their caregiving roles or inherent disparities in perceived health status and well-being.

**Table 7.** Overall evaluation of the quality of life and satisfaction with the health of family caregivers of individuals with rare diseases by sex.

	Male, n (%)	Female, n (%)	Total, n (%)
How would you rate your quality of life?			
Very poor	7 (5.4)	17 (2.1)	24 (2.5)
Poor	19 (14.6)	108 (13.3)	127 (13.5)
Neither poor nor good	41 (31.5)	321 (39.5)	362 (38.4)
Good	55 (42.3)	321 (39.5)	376 (39.9)
Very good	8 (6.2)	45 (5.5)	53 (5.6)
How satisfied are you with your health?			
Very dissatisfied	4 (3.1)	26 (3.2)	30 (3.2)
Dissatisfied	22 (16.9)	237 (29.2)	259 (27.5)
Neither satisfied nor dissatisfied	41 (31.5)	293 (36.1)	334 (35.5)
Satisfied	59 (45.4)	237 (29.2)	296 (31.4)
Very satisfied	4 (3.1)	19 (2.3)	23 (2.4)

Normative data for the 4 dimensions of the WHOQOL-BREF—physical health, psychological health, social relationships, and environment—segmented by sex and age group are presented in Table 8.

Male caregivers demonstrated higher mean scores in physical health than female caregivers across all age groups. The highest mean score was observed in the youngest male group (16‐29 years: mean 59.9, SE 10.04), gradually decreasing with age, reaching its lowest value in the 50+ year group (mean 50.4, SE 4.72). Among female caregivers, the 16‐ to 29-year age group exhibited the lowest mean score (mean 43.6, SE 2.72), while the 40‐ to 49-year age group demonstrated the highest (mean 51.0, SE 1.00). The absence of floor or ceiling effects in this domain indicates that the scale effectively captured a comprehensive range of physical health outcomes without concentrating on the extremes. The observed decline in physical health scores with age is consistent with patterns reported in other populations, suggesting a cumulative impact of caregiving and aging.

Psychological health scores demonstrated relative stability across age groups for both male and female caregivers. Male caregivers exhibited scores ranging from 57.4 to 58.4, while female caregivers displayed scores from 49.5 (16‐29 years) to 56.2 (40‐49 years). Notwithstanding the overall consistency, female caregivers in the 16‐ to 29-year age group reported significantly lower psychological well-being. No ceiling effects were observed, and only minimal floor effects were present (1.1%) among female caregivers aged 50+ years, indicating that extreme values were rare.

**Table 8.** WHOQOL-BREF norms for the Polish population of caregivers of individuals with rare diseases by sex and age.

	Male				Female			
	16‐29 years	30‐39 years	40‐49 years	50+ years	16‐29 years	30‐39 years	40‐49 years	50+ years
Physical health								
Mean (SE)	59.9 (10.04)	58.1 (2.92)	54.0 (2.29)	50.4 (4.72)	43.6 (2.72)	49.5 (0.91)	51.0 (1.00)	45.6 (1.79)
Range	49.9-69.9	21.2-91.5	7.2-92.8	14.5-85.0	17.9-73.7	6.9-92.4	4.8-100.0	7.4-81.7
Median (IQR)	59.9 (54.9-64.9)	60.8 (43.8-72.9)	54.2 (42.9-68.3)	51.6 (33.7-67.4)	44.6 (34.3-52.6)	48.9 (38.7-60.8)	50.0 (38.1-65.6)	47.4 (33.4-57.4)
Floor effect (%)	0	0	0	0	0	0	0	0
Ceiling effect (%)	0	0	0	0	0	0	0.3	0
Psychological health								
Mean (SE)	57.8 (12.33)	58.2 (3.26)	57.4 (2.00)	58.4 (3.98)	49.5 (2.77)	53.6 (0.99)	56.2 (0.96)	52.8 (1.87)
Range	45.5-70.2	7.2-95.5	15.9-91.4	17.9-90.7	21.2-87.7	3.6-95.9	11.7-100.0	0.0-90.7
Median (IQR)	57.8 (51.7-64.0)	63.1 (43.5-71.9)	58.3 (46.1-70.2)	58.0 (48.9-72.3)	49.5 (37.6-60.1)	53.8 (41.4-66.9)	57.7 (45.2-70.4)	54.0 (41.9-66.4)
Floor effect (%)	0	0	0	0	0	0	0	1.1
Ceiling effect (%)	0	0	0	0	0	0	0	0
Social relationships								
Mean (SE)	55.0 (4.75)	52.4 (3.21)	50.8 (2.67)	52.1 (4.82)	39.3 (3.34)	51.2 (1.17)	52.5 (1.05)	50.9 (1.91)
Range	50.2-59.7	7.7-92.3	0.0-100.0	17.3-100.0	0.0-75.0	0.0-100.0	0.0-100.0	0.0-84.5
Median (IQR)	55.0 (52.6-57.3)	57.5 (40.3-67.3)	52.3 (40.9-65.8)	57.4 (33.5-67.3)	41.3 (25.0-50.1)	50.0 (34.7-67.5)	52.2 (40.1-67.5)	52.2 (34.9-67.3)
Floor effect (%)	0	0	4.7	0	6.2	1.2	0.9	1.1
Ceiling effect (%)	0	0	1.6	5	0	1.8	1.1	0
Environment								
Mean (SE)	52.8 (2.02)	57.5 (2.39)	55.3 (1.84)	51.5 (4.09)	48.8 (2.73)	51.1 (0.92)	53.0 (0.93)	48.5 (1.91)
Range	50.8-54.8	24.5-87.2	15.8-83.4	23.0-84.7	14.2-73.3	6.8-97.8	9.5-94.2	9.1-87.8
Median (IQR)	52.8 (51.8-53.8)	59.5 (47.2-68.8)	54.1 (46.3-65.4)	50.2 (34.6-65.4)	48.0 (38.2-61.8)	51.3 (39.8-62.5)	52.0 (41.5-65.5)	50.2 (36.0-62.9)
Floor effect (%)	0	0	0	0	0	0	0	0
Ceiling effect (%)	0	0	0	0	0	0	0	0

a WHOQOL-BREF: World Health Organization Quality of Life-BREF.

The social relationships domain displayed greater variability, particularly among female caregivers. The lowest mean score was found in the youngest female group (16‐29 years: mean 39.3, SE 3.34), suggesting social challenges in this group. In contrast, male caregivers demonstrated more stability, with scores ranging from 50.8 to 55.0 across all age groups. Significant floor effects were observed among female caregivers aged 16‐29 years (6.2%) and male caregivers aged 40‐49 years (4.7%), suggesting that many respondents experienced severe difficulties in social relationships. Ceiling effects were generally minimal, with the highest occurrence (5%) observed among male caregivers aged 50 years and older.

The scores in the environment domain demonstrated greater consistency across age groups. The mean score was highest among male caregivers aged 30‐39 years (mean 57.5, SE 2.39) and lowest among female caregivers aged 50+ years (mean 48.5, SE 1.91). The absence of floor or ceiling effects indicated that responses were distributed uniformly across the scale. This dimension reflects a stable perception of environmental support and resources among caregivers, irrespective of age or sex.

The study’s results indicate significant disparities in HRQoL within the sex and age categories of caregivers. Specifically, younger female caregivers exhibited lower scores in various domains of WHOQOL-BREF. These disparities can be attributed to the unique challenges associated with caregiving roles, personal health, and social support systems, particularly for specific demographic groups.

## Discussion

### Principal Findings

The WHOQOL-BREF is a widely used instrument for assessing QoL across diverse populations [4,28]. Its psychometric properties have been extensively studied, confirming its reliability and validity. However, certain aspects warrant further discussion, including its applicability across cultural contexts, domain-specific reliability concerns, and its use in both general and disease-specific populations [8,9,26,29-32].

Our study demonstrated that the WHOQOL-BREF is an appropriate tool for assessing QoL among caregivers of individuals with RDs. The instrument exhibited good internal consistency, with Cronbach α values generally exceeding 0.70. Internal consistency analysis showed values ranging from 0.69 for the social relationships domain to 0.84 for psychological health, indicating satisfactory to high reliability across domains. The item-level analysis identified specific areas where refinements could enhance reliability. Nevertheless, previous studies have reported lower reliability in the social relationships domain, highlighting a potential need for further refinement [6,8,33].

The construct validity of the WHOQOL-BREF is well established, with CFAs generally supporting its 4-domain structure. The instrument also demonstrates strong convergent and discriminant validity, correlating well with other health measures and effectively distinguishing between groups with differing health conditions [34-37]. Consistent with prior research, our findings confirm that the WHOQOL-BREF generally maintains good internal consistency, with Cronbach α values frequently exceeding 0.70. For instance, one study reported an α of 0.898 for the total score [33].

However, lower α values have often been observed in the social relationship domain, occasionally falling below 0.70. A Thai study reported a Cronbach α of 0.62 for this domain, while the other domains ranged from 0.81 to 0.85 [38]. Similarly, a Greek study found an α of 0.65 for social relationships, with other domains ranging from 0.66 to 0.87 [36]. Several factors may account for this pattern. The social relationships domain comprises only 3 items, which naturally limits internal consistency compared to domains with more items. Additionally, the items assess complex and sensitive aspects of life—such as personal relationships and sexual activity—which may be interpreted differently across individuals, particularly among caregivers of persons with RDs. Cultural, sex-based, and situational factors (eg, emotional exhaustion, social withdrawal, and limited time for interpersonal interaction) may further influence caregivers’ responses, contributing to variability in this domain.

Factor analysis has been widely used to evaluate the underlying structure of the WHOQOL-BREF [6,39]. CFA results indicate that a unidimensional model yields poor fit indices, whereas the 4-domain model significantly improves model fit. Further refinements, including correlated error terms, have led to an optimized model with improved goodness-of-fit measures (*χ*^2^/*df*, CFI, TLI, RMSEA, and SRMR). These findings confirm the structural validity of the WHOQOL-BREF and reinforce its intended dimensional structure [8,32]. Although CFA generally supports the 4-domain model (physical health, psychological health, social relationships, and environment domains), some studies have questioned this structure, reporting that certain items do not clearly discriminate between domains or correlate more strongly with an unintended domain [32,34,36,38]. Exploratory factor analysis in a Norwegian study also resulted in a 4-component solution, partially supporting the established domain structure [8]. Some studies suggest that the physical health domain contributes most significantly to overall QoL [33].

The WHOQOL-BREF has demonstrated strong psychometric properties across various countries and cultures. Research supports its discriminant validity, content validity, internal consistency, and test-retest reliability across different cultural settings, suggesting its suitability for cross-cultural comparisons [4,8,36,38,40]. The instrument has been translated and adapted for multiple languages, including Polish [6], Korean [33], Spanish [35,40], Portuguese [41], Thai [38], Bahasa Indonesia [42], Mongolian [43], Persian [39], Danish [32], Norwegian [8], and French [9]. The translation process generally adheres to rigorous methodologies, such as forward and backward translation and cultural adaptation procedures. Some adaptations have been proposed to enhance cultural relevance, including the incorporation of additional items related to work satisfaction, social activities, and nutrition [36]. While these modifications may improve local applicability, they could also impact comparability across studies [40].

In studies conducted within the general population, the WHOQOL-BREF has been used to establish normative QoL scores, facilitating demographic comparisons [7,9,44]. Factors such as age, sex, education level, and health status have been identified as influential [4,7,9,32,42,44,45]. For instance, some studies suggest that female caregivers report higher social QoL [26,46], while male caregivers tend to report better psychological QoL [7,9,46]. Physical health scores typically decline with age, reflecting the natural progression of age-related health issues [3,4,46,47].

In clinical settings, the WHOQOL-BREF has been validated or used for various disease groups, including cancer, chronic conditions, mental disorders, HIV/AIDS, Huntington disease, Prader-Willi syndrome, hemophilia, neurofibromatosis, rheumatoid arthritis, and spinal cord injury [4,30,31,34,36,48-54]. It serves as a valuable tool for evaluating the impact of illness on QoL and monitoring treatment effectiveness [4,28]. However, some validation studies have raised concerns regarding the internal consistency of the social relationships domain [8,29]. The instrument’s generic nature allows for comparisons across different medical conditions, ensuring broad applicability in health research. Factorial invariance testing has confirmed that the WHOQOL-BREF consistently measures QoL across different patient groups [4,8,34].

Despite its strengths, certain limitations of the WHOQOL-BREF should be acknowledged. The social relationships domain has exhibited weaker reliability in some studies, suggesting a need for refinement or supplementation with additional items. While the environment domain is a notable strength of the instrument, some researchers have recommended incorporating more sensitive response options to improve measurement precision [6,28,29,46].

The WHOQOL-BREF’s reliance on self-report data enhances respondent acceptability but also introduces potential biases, such as social desirability and response tendencies. Adherence to standardized scoring guidelines is essential for ensuring accurate interpretation, and researchers should consider variations in response patterns across different populations [6,29,32].

In our study, QoL domain scores were normalized to a 0‐100 scale. Factor loadings ranged from 0.363 to 0.801, indicating varying contributions of items to their respective constructs. The psychological health domain demonstrated the highest mean score (mean 54.9, SE 0.59), while the physical health domain had the lowest (mean 50.2, SE 0.59). Normality tests showed slight deviations, particularly in the psychological health and social relationships domains, though skewness and kurtosis values remained within acceptable limits. Minimal floor and ceiling effects ensured adequate response distribution. QoL norms were established for a Polish population of caregivers for individuals with RDs, stratified by sex and age group. Male caregivers generally reported higher physical and psychological health scores than female caregivers. Younger female caregivers (16‐29 years) exhibited the lowest scores in several domains, suggesting potential vulnerabilities in social and psychological well-being. These findings highlight disparities related to caregiving burden, sex, and age-related factors.

### Limitations

It is important to consider the limitations of this study before interpreting its results. First, although 942 caregivers replied and completed the survey, the number of caregivers of persons with RDs in Poland is significantly higher. It should be noted, however, that although it can be difficult to extend the results to the total population of caregivers of persons with RDS in Poland, to date, there is no register of individuals with rare conditions in the country. Second, since convenience sampling was used in this survey and the study sample was self-selected, it could further compromise the sample’s representativeness and the results’ generalizability. Third, since caregivers of persons with different RDs took part in the study, there is a possible bias related to challenges related to different conditions, symptoms, and prognoses. However, since most RDs are small in numbers, it would be difficult, if possible at all, to validate the questionnaire for a single RD. Moreover, despite some differences in caregivers’ experiences, numerous previous studies demonstrated that most parents of persons with RDs face similar challenges and express similar needs. Consequently, we believe that the results may still apply to the entire population of family caregivers of persons with RDs in Poland. Especially because the sample is quite diverse, encompassing both mothers and fathers as well as various age groups. Fourth, the self-administering nature of the WHOQOL-BREF is part of its design. However, although the administration may elicit varying degrees of true responses, this bias is consistent across all questionnaires and has no bearing on the accuracy of the psychometric analyses, which are primarily comparative in nature. Fifth, we gathered our data before the Rare Disease Plan for 2024‐2025 was formally approved by the Council of Ministers in August 2024. Sixth, a comparable sample of caregivers of healthy individuals or those experiencing more common chronic diseases could have been beneficial to the study. Seventh, because the study was conducted as a web-based survey, there is a risk that it did not reach caregivers who lived in distant areas without internet access, who were hesitant to use technological devices and social media, or who did not belong to digital associations, foundations, and organizations of patients with RDs or support groups that aided in collecting the data. Consequently, while there is no direct generalizability of the results shown here to other cross-national samples, it is challenging to evaluate bias and the causes of nonresponses when using web-based survey data. Eighth, a potential bias is also present because only 130 fathers responded to the survey. However, it should be explained that while the sample was obtained through convenience sampling, it is well-documented that in the population of family caregivers, there is a significant underrepresentation of male caregivers. Thus, while these sex differences in caregiving could affect how respondents report their QoL, particularly in areas like psychological health and social relationships due to the small number of male respondents, sex-based factor analysis was not possible. Future research should ensure more balanced sex representation and explore sex differences in WHOQOL-BREF performance. Moreover, the 2006 Polish validation of WHOQOL-BREF did not account for sex, age differences, or caregivers of individuals with RDs, highlighting the need for validation in specific populations and subgroup adaptations. Another limitation of this study is the inclusion of caregivers of individuals with 158 different RDs, which prevented detailed clinical characterization of care recipients. However, only some syndromes, that is, Dravet, Angelman, Williams, 22q11DS, and Duchenne or Becker muscular dystrophy, were represented by multiple caregivers; most conditions had only a few or even single respondents. This reflects the nature of rare, ultrarare, and hyperrare disorders, which affect very small patient populations and make it difficult to validate any tool. Future studies should consider focusing on more homogeneous caregiver groups. Next, since no other QoL instrument has ever been validated in the population of family caregivers of persons with RDs in Poland, this lack of external validation constitutes a limitation of our study and highlights the need for future research dedicated specifically to this group. Finally, this study is also limited by the WHOQOL-BREF’s potential insufficiency in capturing social aspects of caregiving; future research should consider using complementary tools like the Zarit Burden Interview.

### Conclusions

The WHOQOL-BREF remains a reliable and valid instrument for assessing QoL across diverse populations. Its 4D structure is robust, and normative data provide valuable benchmarks for assessing QoL in specific groups, such as caregivers. Future research should focus on refining the social relationships domain and addressing minor fit inconsistencies to enhance the scale’s applicability further. An important question arises regarding whether the social relationships domain, due to inherent differences between societies, patient groups, and caregivers of various conditions, can be conceptualized as a universal construct. While the WHOQOL-BREF remains a valuable tool for assessing QoL in diverse populations, careful consideration of item profiles and potential supplementary modules for specific age groups may further optimize its utility.

## Supplementary material

10.2196/72590 Checklist 1 STROBE (Strengthening the Reporting of Observational Studies in Epidemiology) checklist.

## Abbreviations

CFA: confirmatory factor analysis
CFI: Comparative Fit Index
HRQoL: health-related quality of life
QoL: quality of life
RD: rare disease
RMSEA: root-mean-square error of approximation
SRMR: standardized root-mean-square residual
STROBE: Strengthening the Reporting of Observational Studies in Epidemiology
TLI: Tucker-Lewis Index
WHO: World Health Organization
WHOQOL: World Health Organization Quality of Life
WHOQOL-BREF: World Health Organization Quality of Life-BREF
